# Injuries among students combining academic studies and elite sports

**DOI:** 10.3389/fspor.2025.1561279

**Published:** 2025-09-05

**Authors:** Emil Flatholm, Eva Tengman, Taru Tervo

**Affiliations:** ^1^ Department of Community Medicine and Rehabilitation, Section for Physiotherapy, Umeå University, Umeå, Sweden; ^2^ Umeå School of Sport Sciences, Umeå University, Umeå, Sweden

**Keywords:** dual career athletes, student athletes, injuries, injury incidence, elite sports

## Abstract

**Objective:**

The purposes of the study were to examine injury incidence among students engaged in elite sports during their first year of study, and to explore factors associated with injury in this population.

**Methods:**

Two surveys were carried out one year apart. A total of 243 students responded to the initial baseline survey; 111 also responded to a follow-up survey. The surveys addressed injury events and characteristics, sports participation, experienced stress, relaxation, control during leisure time, and study pace.

**Results:**

Prior to commencing their studies, 51% of the athletes had experienced an injury; 54% experienced an injury during their first year of study. A previous injury increased the odds of a new injury (OR = 3.174, *p* < 0.01). Athletes sustaining an overuse injury prior to studies had higher risk for sustaining a new overuse injury during the first year of studies (OR = 4.312, *p* < 0.001), while sustaining an acute injury prior to studies did not significantly increase the risk for sustaining a new acute injury. Sex, type of sport, and study pace were not associated with increased odds of injury.

**Conclusion:**

A concerning 54% injury incidence was found during the first year of study. A previous injury increased the likelihood of sustaining a new injury. The findings suggest that injury-prevention methods should be focused particularly on students who have been injured in the year before they begin their studies.

## 1 Introduction

Dual-career programmes have been implemented by higher-education institutions all over the world to help bridge two separate areas—sports careers and academic studies—and facilitate success in both, while simultaneously laying the foundation for a smooth transition to an alternative career when the athlete's sporting career ends (1, 2). There are several challenges in pursuing dual careers such as the athlete's desire to succeed in both studies and sports adding pressure, a full schedule of lectures and training/competition leaving little room for relaxation, the need to continuously adapt to new environments, financial difficulties, and problems maintaining a rich social life (2). A biopsychosocial approach is recommended for identifying, preventing, and rehabilitating sports injuries (3–5). One third of first-year university students describe mental-health problems due to low academic function in relation to being new to academic studies (6). Linner et al. conclude that the transition from high school to university is a large challenge for student athletes due to environmental changes and higher demands on performance in both studies and sports (7). Academic pressure has been found to increase the occurrence of sports injuries by 9% (8), and students who experience high academic stress are three times more likely to sustain a sports injury than students experiencing low academic stress (9).

It has been found that sports injuries are a widespread problem among university athletes. Kimura et al. found that 50% of 5,500 Japanese collegiate athletes experienced one or more injuries over the course of 1 year (10). Teahan et al. (11) examined injury incidence among 672 university athletes in Ireland, and presented similar results: one in four student athletes sustained an injury over the course of one academic term. Moreover, they found that acute injuries were more common than overuse injuries and that most injuries affected muscles and ligaments (11). The results from these studies differ with regard to gender differences (10, 11). It is well understood that previously sustained sports injuries increase the risk of sustaining new sports injuries (12, 13). Similarly, factors such as negative life events, high levels of life stress, daily hassle, and stress from a previous injury predict an increased sports-injury risk (14). This is further supported by Ivarsson et al.'s finding that negative life-event stress and stress responsivity are associated with an increased risk of sports injury (15).

Participation in sport comes with the risk of sustaining a sports-related injury, and research suggests that being a first-year university student also increases injury risk (8, 9). This, in combination with student athletes describing having insufficient time for both sports and academic studies (16), highlights the importance of examining this population further. Therefore, the primary aim of this study was to examine injury incidence among athletes who combined academic studies and elite sports, both before and during their first year at university. A second aim was to describe the mechanisms behind and types and locations of the injuries that occurred during the students' first year of their studies. The third aim was to examine whether sex, prior injuries, type of sport, psychological stress, and psychosocial factors such as study pace, relaxation, and leisure time were associated with injury during the first year of study.

## 2 Methods

### 2.1 Study design

This prospective cohort study is part of a larger, ongoing project that is being carried out at a university in Sweden; the aim of this project is to examine the outcomes of combining elite sports and university studies by collecting survey data electronically. The main project consists of six cohorts; the first cohort began studying in 2018, and was followed by a new cohort each year thereafter. Each cohort participated in a baseline survey at the start of their studies, and an annual follow-up survey. The data for the present study was gathered from the main project, and includes data from the baseline and one-year follow-up surveys sent out between 2018 and 2022.

### 2.2 Participants

**Figure 1:**
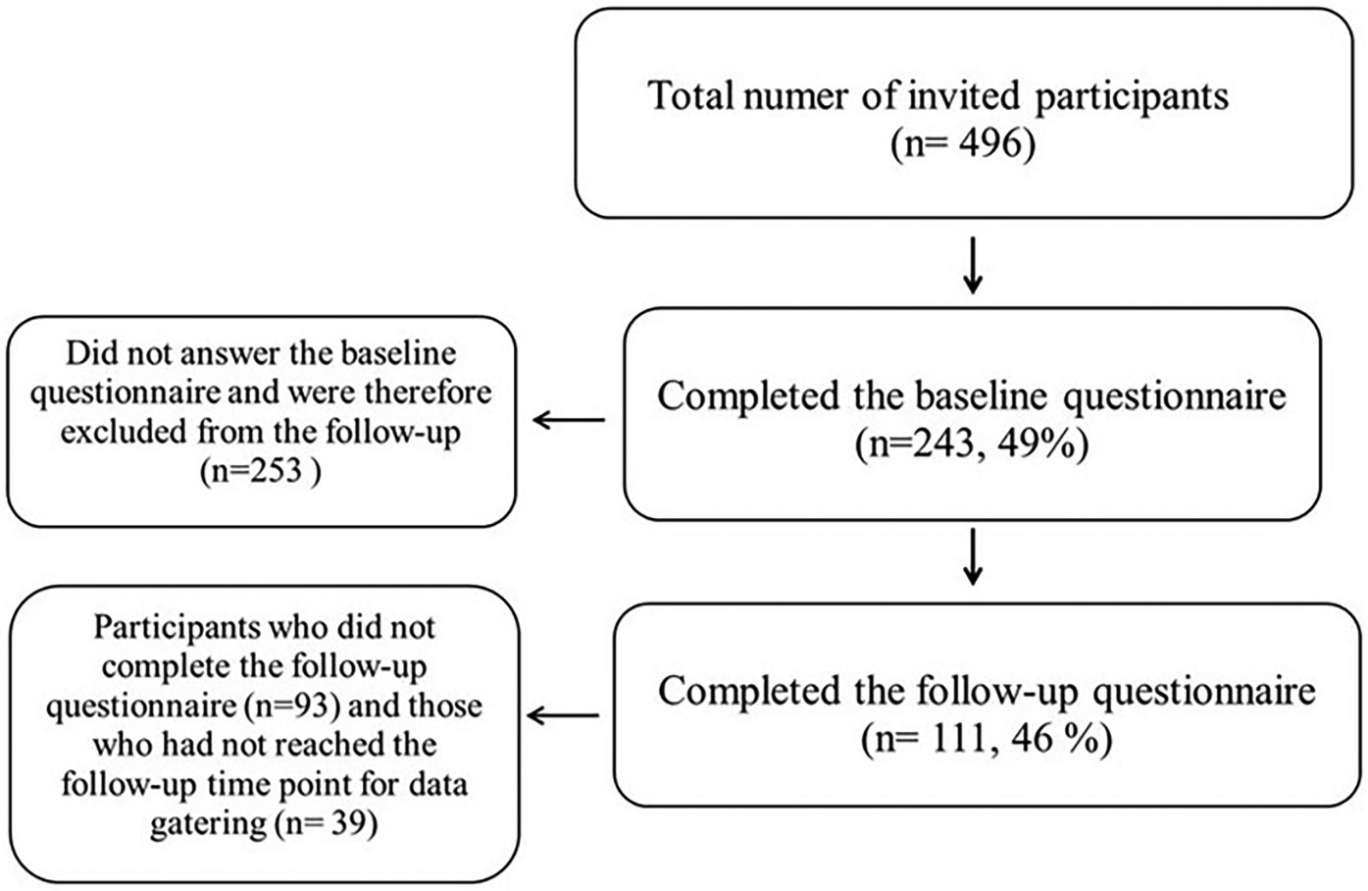
Participant flow diagram. The figure illustrates the number of student athletes invited to participate the study, those who completed the baseline and follow-up questionnaires, and also the number of student athletes who dropped out or did not included during the study period.

The participants in the main project were informed about its purpose, and signed a written consent form. Of the baseline surveys that were sent out, 49% were filled in and returned, amounting to a total of 243. These were included in the present study. A total of 111 (46%) of the 243 participants also answered the 1-year follow-up survey. See the participant Flowchart diagram in Figure 1. See Tables 1, 2 for participant characteristics.

**Table 1:** Participant characteristics.

Variables	Baseline *n* = 243			One-year follow-up *n* = 111			
	Men	Women	Total (%)		Men	Woman	Total (%)
Sex *n*	101	142	243		47	64	111
Age (min-max)	22.4 (19–36)	21.7 (18–34)	–		23.8 (20–37)	22 (19–30)	–
Study pace *n*							
25%	2	3	5 (2)		0	1	1 (1)
26%–50%	11	3	14 (6)		6	4	10 (9)
51%–75%	9	8	17 (7)		4	1	5 (5)
76%–99%	39	40	79 (33)		9	8	17 (15)
100% or more	40	88	128 (53)		28	50	78 (70)
Type of sport *n*							
Individual sport	78	74	152 (63)		43	34	77 (69)
Team sport	23	68	91 (37)		4	30	34 (31)

**Table 2:** Sports practiced by participants.

Sport participated in *n*	Baseline (%) *n* = 243	One-year follow-up (%) *n* = 111
Badminton	16 (7)	5 (5)
Basketball	11 (5)	5 (5)
Soccer	21 (9)	7 (6)
Athletics	23 (10)	15 (14)
Floorball	39 (16)	15 (14)
Hockey	11 (5)	3 (3)
Orienteering	20 (8)	15 (14)
Skiing	57 (24)	27 (24)
Martial arts	10 (4)	4 (4)
Other	34 (14)	15 (14)

A non-response analysis was made to compare the dropouts, *n* = 132, with the subjects that answered the 1-year follow-up, *n* = 111 (see “Supplementary Material”). Of the 132 dropouts, 39 did not yet receive the 1-year follow-up survey at the point of data gathering for the present study. The remaining 93 dropouts chose not to answer the 1-year follow-up survey. There was a significant difference in sports participation; practitioners of individual sports more commonly answered the 1-year follow-up survey than practitioners of team sports (*p* = 0.044; see “Supplementary material”). For all other variables, the 1-year follow-up group was not significantly distinct from the dropout group.

### 2.3 Variables

The background variables were chosen to present the characteristics of the subjects, and originated from existing validated questionnaires (17–19). Questions regarding injuries were created based on the recommendations of Côté et al. for tracing the development of athletes (17). Psychosocial factors were measured using questions from the Recovery Experience Questionnaire (REQ) (18). For this study, two dimensions were analysed: relaxation and control during leisure time. Each REQ question was answered on a 1–5 scale, and the mean values for each dimension, which consisted of four questions, were then calculated in order to be used for the analysis. The REQ translated into Swedish has been shown to have high reliability and validity (20). Experienced stress was measured in the same way as in the Swedish National Public Health Survey (19). Variable stress, which originally had four answer options, was reworked to have two options: “not stressed” (not at all or to some degree) and “stressed” (pretty much or very much) to be sufficient for analysis.

Sex referred to being either a man or a woman. Age was measured in whole years. For the follow-up survey, 1 year was manually added to the age of each subject since the participants did not fill in their age again. There were five answers for study pace, ranging from 25% to more than 100%, but to facilitate the analysis three ranges were constructed: 25%–75%, 76%–100%, and 100% or more. Participants specified which sport they participated in. To ensure anonymity (particularly because some sports had very few participants), the sports were later categorised as either a “team sport” or an “individual sport”. The participants answered regarding whether they had suffered acute and/or overuse injuries in the previous 12 months that had kept them from participating in practice or competition, and described the injury sustained in a text box. An acute injury was classed as one that occurred abruptly, such as a sprain, concussion, or muscle tear, and that hindered participation in sport. An overuse injury was classed as pain that had increased over time, and ultimately hindered participation in sport. A participant stating that they had suffered an acute or overuse injury, or a combination of the two, resulted in them being considered to have suffered from an injury in the previous 12 months. Using the text boxes filled in by the participants, additional variables were constructed that described e.g., in which region of the body the injury occurred, and which structure it affected. Injury locations were categorised as the upper body, the lower body, the head, or a combination (two or more areas during the same event). The type of injury was categorised as muscular, joint/ligament, skeletal, concussion, a combination (two or more injury types during the same event), or other. In text boxes, after indicating whether the injury was acute or overuse, not every participant presented sufficient information regarding what structure was injured (37 responses) or the location of the injury (22 responses). Only answers that were clearly stating what structure was injured and what body part was affected were added to the analysis. The software used to read the baseline surveys did not interpret and code every answer correctly, which led to a small rejection of data (10 responses).

### 2.4 Statistical analyses

The Jamovi software package for desktop, Version 2.3 (21) and Statistical Package for the Social Sciences, version 28 (IBM SPSS, Armonk, New York, USA), was used for analyses. Exploratory descriptions and frequencies were used to present the characteristics of the baseline, dropout, and 1-year follow-up groups. The dropout and 1-year follow-up groups were compared in a non-response analysis to identify any differences. Differences between the groups in terms of frequencies were analysed using the chi-square test, and an independent samples *t*-test was conducted to examine the differences in means between the groups. Injury incidence in the 12 months before the beginning of studies and during the first year of studies was presented in frequencies and, in text, converted to percentages. Differences in injury incidence by prior injury, sex, and sport were analysed using the chi-square test. Descriptive frequencies were used to present the types and locations of injuries, while differences in terms of injury mechanism in relation to sex and type of sport were analysed via the chi-square test. Experienced stress and REQ means were compared between injured and non-injured students with chi-square test and independent samples *t*-tests. Univariate logistic regressions were conducted to examine whether the type of sport participated in, sex, and study pace affected the odds of sustaining a sports injury. Relation between acute and overuse injuries prior to studies to the first year were analysed via the chi-square test with an odd ration estimate. Alpha was set to 0.05 for all analyses.

## 3 Results

### 3.1 Injury incidence at baseline and during the first year of studies

The injury incidence for the whole group was 51% (124 injured; 119 injury-free) in the 12 months before baseline and 54% (60 injured; 51 injury-free) during the first year of university studies. A total of 69 injuries were reported during the first year of studies in 60 students. Most injuries (77%) occurred in individual sports (see Table 3). However, no significant differences in injury incidence were seen between individual and team sports athletes nor between men and women.

**Table 3:** Injury incidence 12 months before baseline, and during the first year of studies. Chi-square analysis for differences in injury incidence between sexes and between sports.

	Men	Women	Total n	*P*-value	Team sport	Individual sport	Total n	*P*-value
Injury suffered in the past 12 months (before baseline)								
Yes *n* (%)	51 (41)	73 (59)	124		50 (40)	74 (60)	124	
No *n* (%)	50 (42)	69 (58)	119	0.888	41 (33)	78 (66)	119	0.345
Injury suffered in the first year of studies								
Yes *n* (%)	28 (47)	32 (60)	60		14 (23)	46 (77)	60	
No *n* (%)	19 (37)	32 (66)	51	0.38	20 (39)	31 (61)	51	0.07

### 3.2 The first year of dual-career studies—injury mechanism, type, and location

Overuse injuries were the most common injury mechanism (59%; 41 overuse injuries, 28 acute injuries) during the first year of studies (see Table 4).

**Table 4:** Mechanism of injury for participants injured during their first year of studies. Chi-square analysis for differences in injury mechanisms between sexes and type of sport participated in.

Variable	Men	Women	Total *n*	*P*-value	Individual sport	Team sport	Total *n*	*P*-value
Acute injury								
Yes *n* (%)	13 (46)	15 (54)	28*		20 (71)	8 (29)	28	
No *n* (%)	34 (41)	49 (59)	83	0.613	57 (78)	26 (22)	83	0.79
Overuse injury								
Yes *n* (%)	20 (49)	21 (51)	41*		32 (78)	9 (22)	41	
No *n* (%)	27 (39)	43 (61)	70	0.293	45 (64)	25 (36)	70	0.13

* Total amount of injuries ≠ 60 injuries due to some participants reporting both a acute and overuse injury.

As can be seen in Table 5, the type of injury most commonly sustained during the first year of studies was joint/ligament injury (52%), followed by muscular injury (26%). Most of the injuries during the first year of study occurred in the lower body (55%).

**Table 5:** Types and locations of injuries sustained during the first year of studies.

Variables	Total *n* (%)
Type of injury^a^	*n* = 23
Muscular	6 (26)
Joint/ligament	12 (52)
Skeletal	1 (4)
Combination	4 (17)
Injury location^a^	*n* = 38
Upper body	12 (32)
Lower body	21 (55)
Combination	5 (13)

^a^ Not every participant presented information sufficient to distinguish the type of injury or injury location.

### 3.3 Experienced stress and psychosocial factors associated with injuries during the first year of studies

Table 6 shows that there were no significant differences in terms of stress levels at the time of measurement, and REQ mean values between those who suffered an injury and those who did not during the first year of their studies.

**Table 6:** Experienced stress, relaxation, and control during leisure time during the first year of studies for injured and non-injured student athletes.

Variable	Injured *n* = 60		Non-injured *n* = 51		
	*N*	95% CI	*n*	95% CI	*P*-value
Experienced stress					0.90
Not stressed *n* (%)	40 (67)	–	34 (67)	–	
Stressed *n* (%)	19 (23)	–	17 (23)	–	
REQ control dimension mean (SD)	3.96 (0.726)	3.77–4.15	3.99 (0.704)	3.79–4.18	0.87
REQ relaxation dimension mean (SD)	3.53 (0.876)	3.31–3.76	3.47 (0.820)	3.24–3.70	0.68

CI, 95% confidence interval; SD, standard deviation; REQ, recovery experience questionnaire.

### 3.4 Recurring acute and overuse injuries

Univariate logistic regressions (see Table 7) showed that sustaining an injury in the 12 months prior to studies increased the odds of sustaining a new injury during the first year of studies (OR = 3.217, *p* < 0.001), while sex, sport and study pace did not significantly change the odds of injury. Those students who had an overuse injury 12 months prior baseline had an increased odds ratio for an overuse injury also during the first year of the study (OR = 4.312, *p* < 0.001), while an acute injury prior baseline did not significantly change the odds of an acute injury (see Table 8).

**Table 7:** Univariate logistic regression analysis of factors affecting the odds of sustaining an injury during the first year of studies.

Factors	OR	95% CI	*P*-value
Sex			
Women vs. men	1.474	0.688–3.16	0.32
Type of sport			
Individual sport vs. team sport	2.120	0.933–4.82	0.07
Study pace			
76%–100% vs. 25%–75%	1.167	0.288–1.73	0.83
100% or more, 25%–75%	1.583	0.524–1.78	0.42
Injury suffered			
Injury at 1-year follow-up vs. injury in the 12 months before baseline	3.217	1.474–7.025	**<0** **.** **01**

OR, odds ratio; CI, 95% confidence interval.

Boldface indicates significance.

**Table 8:** Recurring acute an overuse injuries. Injury mechanism sustained within 12 months before baseline and in the first year of studies. Chi-square analysis for recurring injury mechanism.

Injuries	Yes *n* (%)	No *n* (%)	OR	95% CI	*P*-value
Acute injury			1.871	0.740–4.730	0.185
Baseline	29	82			
Follow-up	28	83			
Overuse injury			4.312	1.877–9.906	**<0** **.** **001**
Baseline	39	72			
Follow-up	41	70			

Amount of injuries ≠ 60 due to some participants sustaining both a acute and a overuse injury.

Boldface indicates significance.

## 4 Discussion

The major finding of this study is that similar rates of injury were seen in the 12 months prior to the start of university studies (51%) and during the first year of university studies (54%). This in contrast to prior research, which reported a yearly injury incidence of 25%–50% in university athletes (10, 11), and underlines the high injury rates in student athletes. Students who sustain an injury prior to their studies are more likely to sustain a new injury in line with prior research showing that previous injury increases the risk of sustaining a new injury (12, 13). It was found that athletes sustaining an overuse injury prior to studies had higher odd ratio for sustaining a new overuse injury during the first year of studies (OR = 4.312), while sustaining an acute injury prior to studies did not significantly increase the risk for sustaining a new acute injury. No statistically significant differences could be seen in injury incidence between the sexes nor between individual and team sports athletes, even though a trend indicating that injuries are more common within individual sports was observed. Our findings regarding no sex differences are in contrast with previous studies on university athletes, which have reported differences in sports injuries between males and females (10, 11, 22). However, while previous studies predominantly included participants from team sports, our study primarily involved athletes in individual sports.

Regarding the high incidence of injuries among student athletes, our study aligns with the results from Kimura's cross-sectional study of Japanese collegiate athletes (10). However, Kimura's study shows that a significantly higher proportion of first-year athletes stayed injury-free, while a significantly higher proportion of second-year athletes or above reported sustaining an injury. This discrepancy in the results can be attributed to differences in training hours, type of sport, varying average ages of study participants, or anthropometry (10). Overuse was the most common mechanism of injury (59%) during the first year of university studies. This deviates from previous findings, where the incidence of overuse injuries varies between 16% and 29%, while acute injury rates vary between 71% and 89% (23, 24). This could be the result of an overrepresentation of participants in individual sports (*n* = 77) in relation to participants in team sports (*n* = 34) in the present study. Overuse injuries have been found to be 3.28 times more likely in university athletes than in high-school athletes (25). This may be the result of the increased demands in terms of both sport and academia when competing and studying at a more advanced level (7). However, the analysis showed no significant overrepresentation of overuse nor acute injuries for either sex or in relation to the different types of sport participated in.

Acute injuries are known to be more common in sports that involve high speeds and contact sports, while overuse injuries are known to be more common in aerobic sports with continuous, repetitive movements (26). Athletes who participate in cross-country skiing, athletics, and orienteering were overrepresented in the present study, and this may explain why overuse injuries were found to be more common. One quarter of the participants in this study were cross-country skiers, and other studies show that cross-country skiers are more prone to overuse injuries than to acute injuries (27). This overrepresentation may have been due to the geographical location of the study, which provides good conditions for winter sports. There were also approximately twice as many woman as men in the present study, which may have affected the results as overuse injuries as more common among women than men (28, 29). In the present study, no differences between sexes were seen regarding injury incidence.

The reported injuries mostly affected joints and ligament structures. This diverges from Teahan et al.'s findings on Irish student athletes, where 53% of injuries in student athletes were to muscles and 24% to ligaments, mostly in the lower extremity (11). More research is needed regarding how the type of sport participated in and sex affect the type of injury suffered in students who participate in elite sports. In the present study, most of the injuries reported for the first year of studies were in the lower half of the body. These findings are in line with other research, in which lower-body injuries represent majority of all injuries sustained (25, 30). This is valuable knowledge for future implementation of preventative measures, however, future studies would benefit from structuring a survey that more precisely asks what structures have been injured as well as what body part was affected.

When identifying, preventing, and rehabilitation of sports injuries, a biopsychosocial approach is suggested (3–5). A recently published consensus statement by Traneus et al. describes that intra- and interpersonal factors, as well as sociocultural factors, are psychosocial risk factors for overuse injuries while stress responses are the strongest psychological risk factor for acute injuries (3). Although not statistically significant in the present study when comparing injured and non-injured athletes, high stress levels are known to increase the risk of injury in sports (5, 14, 15, 31). Previous research has found that negative life stress increases the risk of injury (5, 31). One explanation for this increased risk of injury when stressed is neurological changes in the function of the brain, resulting in slower decision-making (32). Wilkerson et al. found that a slower reaction time increases the risk of sprains and strains in the lower extremity (33). Although it was not a focus of this study, it is well understood that there are increased demands when starting to study at university (6, 7), and it has been found that the transition from high school to university is demanding and negatively affects the mental health of one third of first-year students (6). An increased study pace can result in high experienced academic stress, which has been shown to increase the risk of sustaining a sports injury (8) up to threefold as compared to experiencing low academic stress (9). Previous studies have shown that being an student athlete comes with several challenges such as limited leisure time, overload feelings, abscense from classes and financial uncertainties (34, 35). Stress at the time of measurement, REQ relaxation, and REQ control of leisure time, did not significantly differ between injured and non-injured students in the present study. The dual-careers programme aims to provide the support necessary to handle the stress that comes with starting studies at a higher academic level. Previous research shows that attending a sports-school resulted in satisfaction with academic support, having higher general and sports specific recovery than stress, and having social support (36). However, stress in this study was evaluated at the time of measurement and did not take into account variation in stress-levels more frequently during the first year of studying. It has been found that athletes are more prone to stress during the pre-season as well as during exam-periods (8) and further research would therefor benefit from measuring stress more often during the year to examine how stress-full periods affect the injury risk in student athletes participating in dual-career programs. More research is needed on how dual-career programmes affect the stress levels of athletes in relation to risk of sports injury.

To date, no previous study has examined the incidence of injuries among student athletes in a Swedish setting, including even athletes in winter sports. The results of our study are consistent with previous research, indicating that student athletes are at high risk of sustaining injuries. Our study highlights that, in university settings, the risk of sustaining a new injury during the first year is particularly high for students who have had a prior injury. Since injuries leads to time off from sports or even ends an athletic career, which in turn may threaten the psychosocial well-being of student athletes, preventive measures in university settings are highly warranted.

### 4.1 Implications for future research and limitations

Methodologically, the present study has both strengths and limitations. A non-response rate of 49% at baseline and 46% at the 1-year follow-up may have affected the credibility of the study. The nonresponse analysis (see in Supplementary Table) showed that the participants in individual sports answered the 1-year follow-up survey to a greater extent than participants in team sports. This may explain why overuse injuries were more common in the examined population. As discussed, women and individual sports practitioners were overrepresented in this study which may be a reason to why overuse injuries and injuries within individual sports were the most common These factors may have influenced the results and limit the generalizability of the findings for male athletes and those involved in team sports in this population. Therefore, in future studies, a larger sample and more even distribution of participants in terms of sexes, individual sports, team sports, and specific sports are recommended.

Another limitation was that the software used to read the baseline surveys did not interpret and code every answer correctly, which led to a small rejection of data. The survey questions also had limitations. The text boxes for detail regarding sustained injuries were not always completed in detail or clearly, which resulted in the author having to reject answers that were not comprehensible in order to avoid misinterpreting them. Regarding the questions about acute and overuse injuries, the participants were asked to think back on injuries suffered in the past year, which can lead to recall bias. However, Gabbe et al. examined the validity of self-reported 12-month injury history, and showed that 80% of participants were able to accurately recall the number of injuries and regions of the body injured, but only 61% could recall the diagnosis (37). Future research would benefit from regularly measuring injury occasions over the year as well as continuing measures over more terms. The experienced stress was measured in the same way as in the Swedish National Public Health Survey (19) and psychosocial factors were measured using the REQ which has been shown to have high reliability and validity (20) which strengthen the methodology. The participants were asked about their experience of stress when they were completing the survey, and it can be argued that this type of question only reflects stress levels for that moment in time. This may be a limitation since stress may vary over the year and be impacted by several factors such as pre-season loads and examination periods, which may have affected the result. Future research is there for recommended to take this into account and measure stress levels more frequently over the year, especially during periods more prone to increase stress as described above.

In the present study, various factors associated with the risk of injury were explored. A methodological limitation was the sample size. A larger sample size would allow for more robust and comprehensive multivariate analyses.

It would be beneficial in future research to compare student athletes involved in dual-career programs with students without involvement in dual-career programs to evaluate if being part of a dual-career program impact the risk for sustaining an injury. The present study focus one the first year of studies, future studies with are recommended to extend the follow-up time and to include other important factors, such as training load, sleep quality, nutritional habits.

## 5 Conclusions

This study highlights a concerning 54% injury incidence during the first year of student athletes' university studies. This underscores the ongoing challenge of sports injuries in this context. The students who had sustained an injury in the 12 months prior to commencing their studies were more prone to sustaining a new injury during their first year of studying. Athletes who sustained an overuse injury prior to the study had a higher odds ratio for sustaining a new overuse injury, while an acute injury prior to the study did not significantly increase the risk of sustaining a new acute injury. A trend of injuries being more common within individual sports than team sports in the given settings was observed. To further examine, studies in the future would benefit from measuring stress more regularly, especially in periods commonly related to high stress such as during the athletes' pre-season or during examination periods. More research is required in order to understand how dual commitments and dual-career programmes impact the likelihood of injury in elite athletes who are studying, and to provide knowledge about and guidance regarding targeted preventative measures.

## Data Availability

The datasets presented in this article are not readily available because the small sample size of the sports increases the risk of identifying the athletes. Requests to access the datasets should be directed to the corresponding author, taru.tervo@umu.se.
