# Supplementary material for: Injuries among students combining academic studies and elite sports
Source: Front Sports Act Living. 2025 Sep 5;7:1561279. doi: 10.3389/fspor.2025.1561279 (PMC12446360; doi:10.3389/fspor.2025.1561279)
Supplement: Supplementary file 1 [file Table1.docx]

**Supplementary material**

Non-response analysis for the subjects that answered the one-year follow-up survey, and that did not answer the one-year follow-up survey (i.e. dropouts).

| **Variables** | **Dropouts**  **n = 132** | **One-year follow-up**  **n = 111** | **P-value** |
| --- | --- | --- | --- |
| Participants n | 132 | 111 |  |
| Man/Woman n | 54/78 | 47/64 | 0.821 |
| Injury in the 12 months before baseline yes/no n | 70/62 | 54/57 | 0.496 |
| Individual sport/team sport n | 75/57 | 77/34 | **0.044*** |
| Study pace |  |  | 0.635 |
| 25–75% n | 23 | 13 |  |
| 76–100% n | 40 | 39 |  |
| 100% or more n | 69 | 59 |  |
| Experienced stress |  |  | 0.362 |
| Not stressed n | 100 | 79 |  |
| Stressed n | 31 | 32 |  |
| Age mean (SD) | 22.1 (3.07) | 21.9 (2.86) | 0.662 |
| REQ – Control mean (SD) | 3.79 (0.911) | 3.88 (0.665) | 0.717 |
| REQ – Relaxation mean (SD) | 3.25 (0.986) | 3.50 (0.740) | 0.364 |
| ***p=<0.05. Comparison between groups by chi-square for n and independent sample t-test for means. REQ: Recovery Experience Questionnaire.** | | | |
